# A multiscale experimental analysis of mechanical properties and deformation behavior of sintered copper–silicon carbide composites enhanced by high-pressure torsion

**DOI:** 10.1007/s43452-021-00286-4

**Published:** 2021-08-17

**Authors:** Szymon Nosewicz, Piotr Bazarnik, Melanie Clozel, Łukasz Kurpaska, Piotr Jenczyk, Dariusz Jarząbek, Marcin Chmielewski, Barbara Romelczyk-Baishya, Malgorzata Lewandowska, Zbigniew Pakieła, Yi Huang, Terence G. Langdon

**Affiliations:** 1 Institute of Fundamental Technological Research, Polish Academy of Sciences, 5B Pawinskiego, 02-106 Warsaw, Poland; 2 Warsaw University of Technology, 141 Woloska Str, 02-507 Warsaw, Poland; 3 National Centre for Nuclear Research, 7 Soltana Str, 05-400 Otwock/Swierk, Poland; 4 Lukasiewicz Research Network, Institute of Microelectronics and Photonics, Center of Electronic Materials Technology, 133 Wolczynska Str, 01-919 Warsaw, Poland; 5 Department of Design and Engineering, Faculty of Science and Technology, Bournemouth University, Poole, Dorset BH12 5BB UK; 6 Materials Research Group, Department of Mechanical Engineering, University of Southampton, Southampton, SO17 1BJ UK

**Keywords:** Copper–silicon carbide composite, High-pressure torsion, Metal–matrix composites, Multiscale analysis, Nano-indentation, Small punch test

## Abstract

Experiments were conducted to investigate, within the framework of a multiscale approach, the mechanical enhancement, deformation and damage behavior of copper–silicon carbide composites (Cu–SiC) fabricated by spark plasma sintering (SPS) and the combination of SPS with high-pressure torsion (HPT). The mechanical properties of the metal–matrix composites were determined at three different length scales corresponding to the macroscopic, micro- and nanoscale. Small punch testing was employed to evaluate the strength of composites at the macroscopic scale. Detailed analysis of microstructure evolution related to SPS and HPT, sample deformation and failure of fractured specimens was conducted using scanning and transmission electron microscopy. A microstructural study revealed changes in the damage behavior for samples processed by HPT and an explanation for this behavior was provided by mechanical testing performed at the micro- and nanoscale. The strength of copper samples and the metal–ceramic interface was determined by microtensile testing and the hardness of each composite component, corresponding to the metal matrix, metal–ceramic interface, and ceramic reinforcement, was measured using nano-indentation. The results confirm the advantageous effect of large plastic deformation on the mechanical properties of Cu–SiC composites and demonstrate the impact on these separate components on the deformation and damage type.

## Introduction

Metal–matrix composites (MMCs) are an important class of materials in which the microstructure can be tailored to have superior properties by comparison with the non-reinforced alloys, including: enhanced high-temperature performance, high specific strength and stiffness, increased wear resistance, better thermal and mechanical fatigue and creep resistance. Due to their possible applications in the aerospace, automotive, defense and general engineering industries, which require durability and long-term performance, the mechanical behavior of the MMCs is a crucial issue dominating their development.

The most significant fabrication methods, such as hot pressing [1], hot isostatic pressing [2], self-propagating high-temperature synthesis [3], pulsed and spark plasma sintering (PPS, SPS) [4] and arc-melting [5], are based on the use of powder metallurgy techniques. However, the fabrication of composites with extraordinary properties via powder metallurgy has several limitations, including the formation of oxide layers around the metal powder particles and the clustering of particles within the material [6] so that further processing is frequently required to improve their mechanical performance. One possible alternative is to apply severe plastic deformation (SPD) as the complementary technique. Thus, SPD procedures have been developed to refine the grain size in metals down to the ultrafine regime below 1 µm and thereby to enhance the mechanical strength [7].

High-pressure torsion (HPT) is an SPD technique that incorporates the application of very high strains and is generally considered the most efficient procedure for achieving grain refinement and strength improvement for several types of materials, such as inter-metallics [8], aluminum alloy [9], titanium-based composites [10] or magnesium alloy [11]. In this technique, a sample is subjected to high applied pressure of typically several GPa, together with concurrent torsional straining. The use of very high pressure prevents the development of cracking and segmentation even in hard-to-deform materials [12] and therefore this procedure is attractive for use in fabricating MMCs. In practice, HPT may enhance the strength of MMCs through the reduction in grain size as well as by an improved homogenization of the ceramic particles [6, 13] caused by the occurrence of some or all strengthening mechanisms at a lower scale of the composite [14].

In practice, the main obstacle to the industrial application of Cu–SiC composites lies in the dissolution of silicon and carbon in the Cu matrix during sintering at elevated temperatures [15, 16], since this strongly influences the thermal and mechanical properties. Processing by HPT is generally conducted at room temperature and some experimental results are already available, confirming the ability to use HPT for the processing of Cu–SiC composites [13, 17, 18].

The HPT processing under a pressure of 10 GPa and 15 revolutions was effective to achieve a complete fragmentation of SiC particles, down to ultrafine particle size in Ref. [17]. HPT method of Cu and Cu–SiC composites enhanced the mechanical properties (hardness and tensile strength) while conserving a reasonable degree of ductility. Furthermore, the tensile yield strength of samples was predicted successfully by the combination between the effect of the processing conditions and the microstructure characteristics through mathematical models. In Ref. [18], metal–matrix composites of Cu and SiC were synthesized by standard HPT and a new technique called high-pressure double torsion (HPDT). Based on detailed microstructural examinations, it was found that significant homogeneity of fine particle distribution as well as weakness of crystallographic texture was provided by HPDT, mainly due to imposing of the highest strain levels.

Finally, standard HPT was employed in Ref. [13] to enhance the structural, mechanical and thermal properties. As was the case with previous investigations, HPT processing led to an improved densification of the SPS-produced composites with significant grain refinement in the copper matrix and with partial fragmentation of the SiC particles and their homogeneous distribution in the copper matrix. Moreover, it was found that processing by HPT also had a major influence on the thermal conductivity of Cu–SiC composites.

Although they show substantial progress in mechanical properties enhancement of Cu–SiC composites, the results provide no quantitative evaluation of the strengthening mechanisms at the micro- or nanoscale. By contrast, the multiscale analysis considers the results at different length scales and combines the efficiency of the macroscopic approach with data collected at the microscopic and nano-levels. For example, nano-indentation testing is an example of a fine-scale experimental technique that provides insight into the nanoscale mechanical behavior of each composite component [19].

**Fig. 1:**
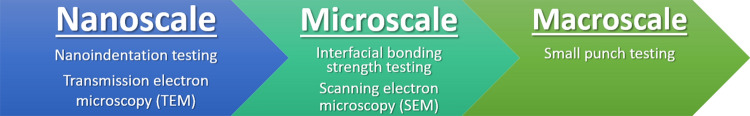
The scope of the experimental multiscale framework of microstructural and mechanical characterization of Cu–SiC composites

The present research shows the first multiscale investigation of the mechanical properties of MMCs processed by HPT where the emphasis is placed on the improvement in the mechanical performance of the Cu–SiC composites. The effect of HPT processing on metal–matrix composites has been revealed by the application of a multiscale experimental framework at the three different length scales (*macroscopic, microscopic, nano-scale*) consisting of several microstructural and mechanical testing approaches (Fig. 1). The presented work focuses on the evolution of properties of the three composite components—metal matrix, ceramic reinforcement and interface zone at metal–ceramic bonding area, and their impact on the composite itself. Moreover, the application of the multiscale approach makes it possible to describe and explain the change of deformation and damage character of Cu–SiC composite subject to HPT in comparison to SPS one. The proposed framework has been presented in detail in the section below.

## Experimental materials and procedures

### Manufacturing and microstructural characterization

**Fig. 2:**
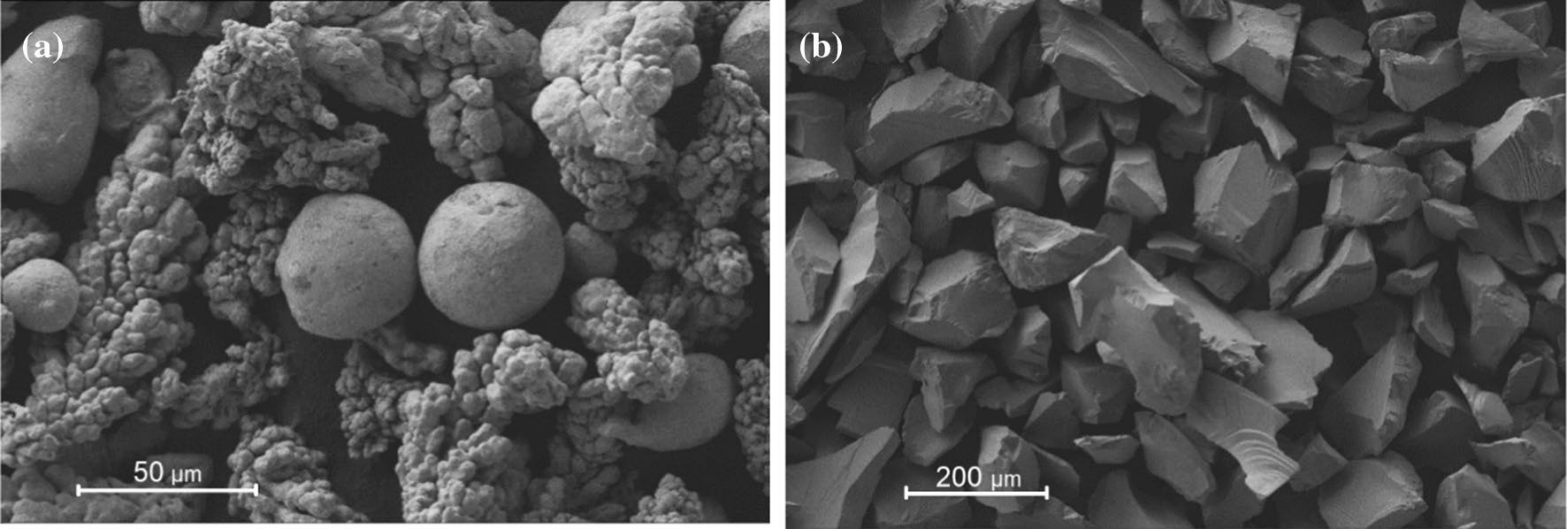
The morphology of the initial powders: **a** copper and **b** silicon carbide [16]

Copper powder having a size of < 40 μm and 99.99% purity (NewMet Koch) and SiC particles with a mean size of ~ 80 µm and 99.99% purity (Saint-Gobain) were used as the raw materials (Fig. 2). Specimens containing 10 and 20 vol% SiC particles were prepared using mechanical mixing in a planetary ball mill for 2 h with a rotational speed of 100 rpm and a ball-to-powder ratio of 5:1. Copper and silicon carbide powder were simultaneously placed in a 250 ml mixing container made of tungsten carbide and cobalt. For the mixing process, 10 mm diameter balls (WC + Co) were used.

The first step of material densification was the sintering of the powder mixtures using a SPS device self-designed and self-constructed in Łukasiewicz-IMIF within a vacuum chamber at a level of 5 × 10^–5^ mbar. The graphite die with the internal diameter of 11 mm and the external diameter of 28 mm were used to obtain the final samples (∅10 × 10 mm). To separate the powder and the die, graphite foil (0.5 mm thick) was applied. To obtain well-densified materials, the sintering temperature was 950 °C with a dwell time of 10 min, a heating rate of 100 °C/min and a compaction pressure of 50 MPa. The temperature of samples was controlled by a thermocouple placed inside the graphite die. Next, the densities of s were measured with the application of the Archimedes method. The obtained results were compared with theoretical densities estimated from the rule of mixtures.

After the initial sintering, the samples were subjected to HPT processing. Disks with a thickness of 1 mm and diameter of 10 mm were subjected to HPT processing under an applied pressure of 6.0 GPa and rotating the lower anvil at 1 rpm through 20 revolutions at room temperature. The applied parameters of HPT process should ensure full saturation effect during deformation (with no further straining and grain size reduction) while simultaneously obtaining homogeneous microstructure in the whole sample volume [13]. This processing was conducted under quasi-constrained conditions which allowed for a small outflow of material around the periphery of the disk during the operation. Henceforth in the paper, the sintered samples are denoted as SPS (Cu SPS and Cu–SiC SPS), and samples manufactured by combination of sintering and high-pressure torsion as HPT (Cu HPT and Cu–SiC HPT).

Both SPS and HPT-processed samples were subjected to microstructural analysis testing. Most structural and fractography studies were conducted using a scanning electron microscope (SEM) Hitachi SU-8000 operating at 10 kV and equipped with a backscatter electron detector (BSE). Detailed observations were performed using a high-resolution scanning transmission electron microscope (STEM) (Hitachi HD-2700). All SEM and STEM microstructural observations were conducted in the periphery regions (approximately 1.5 mm from the edge) for each disk. Thin foils with a thickness of ∼85 nm for STEM observations were extracted from the peripheral regions of each disk using a focused ion beam (FIB) system Hitachi NB 5000.

### Multiscale mechanical characterization

*At the macroscopic scale*, the mechanical properties of SPS and HPT composite samples have been determined via a small punch test (SPT). One of the main advantages of the SPT method is that it requires a small volume of investigated material (mostly in the shape of disks) to obtain a mechanical response. It makes SPT very useful in comparison to other mechanical tests in e.g., tensile or fracture toughness, especially in the case of HPT sample, which demonstrates the relatively small dimensions due to large plastic deformations.

**Fig. 3:**
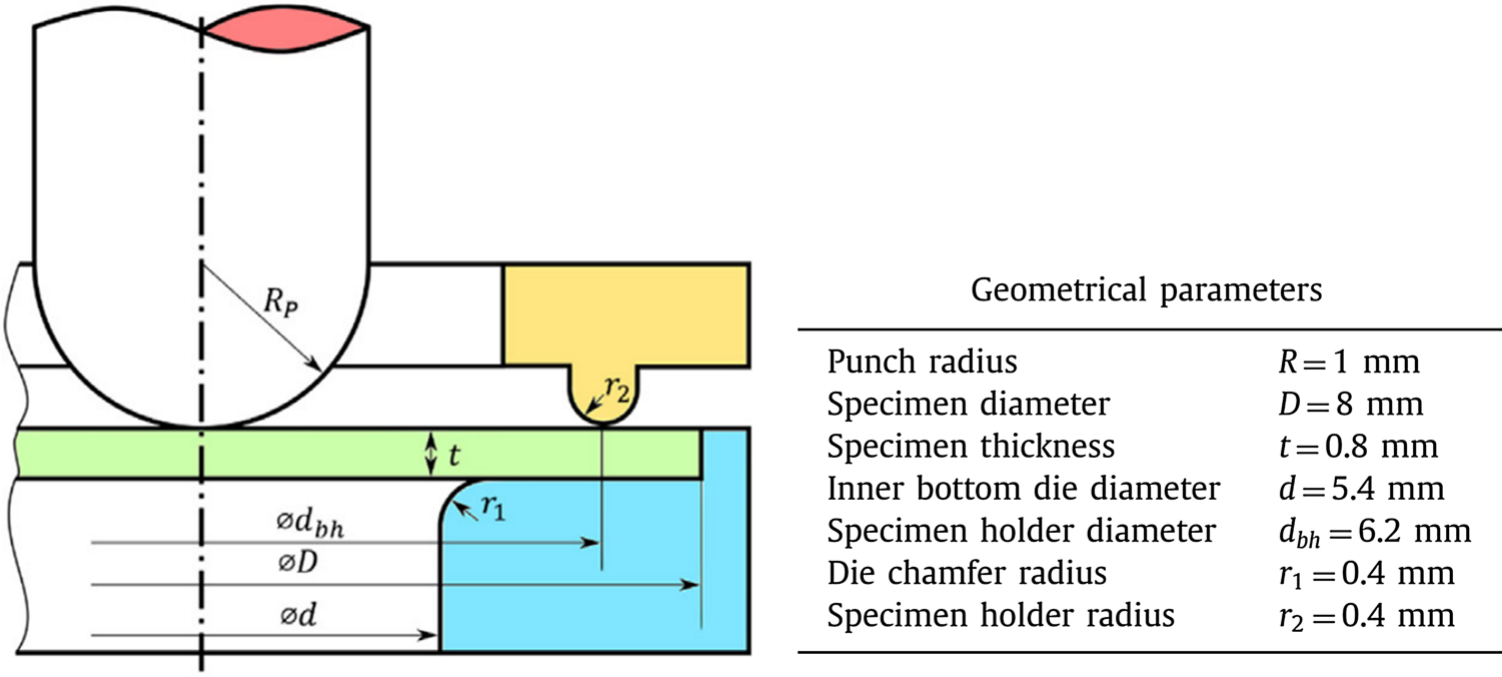
Principle sketch of the experimental set-up of small punch test

The basic scheme of SPT is shown in Fig. 3. In the presented work, the testing was performed at a universal Zwick/Roell Z005 testing machine with a 5 kN load cell and a punch displacement rate of 2.0 mm/min. An electromechanical extensometer MTS 634-12F-25 was applied for measurements of the material deflection using disk-shaped specimens with diameters of 8 mm and heights of 0.6 mm. The SPT samples were cut off from the middle of the HPT disk. For each material state, 3–5 disks were investigated. The testing temperature was 25 °C (marked as RT) and 350 °C measured by a thermocouple located in the testing stand. For higher temperatures, the samples were held 10 min at the designed temperature before SPT. The samples were mounted between two dies, which were further immobilized by a screw. The nut was screwed to a sleeve, with 15 Nm torque. For sample deformation, a spherical punch of 1 mm radius was used and the results of SPT were recorded as force–displacement curves for the punch. The ultimate force (*F*u) was recorded as the maximum registered force.

The application of a biaxial state of tension by bending the miniature disk-shaped specimens causes a complex stress condition usually making it impossible to recalculate stress and strain directly from the register data. However, since the dimensions of samples are presented, we found it valuable to use SPT in the proposed work (similarly as Refs. [20, 21]) for the comparison between two states of materials (SPS and HPT) using force and deflection curves and thus show the impact of HPT on macroscopic composite properties.

**Fig. 4:**
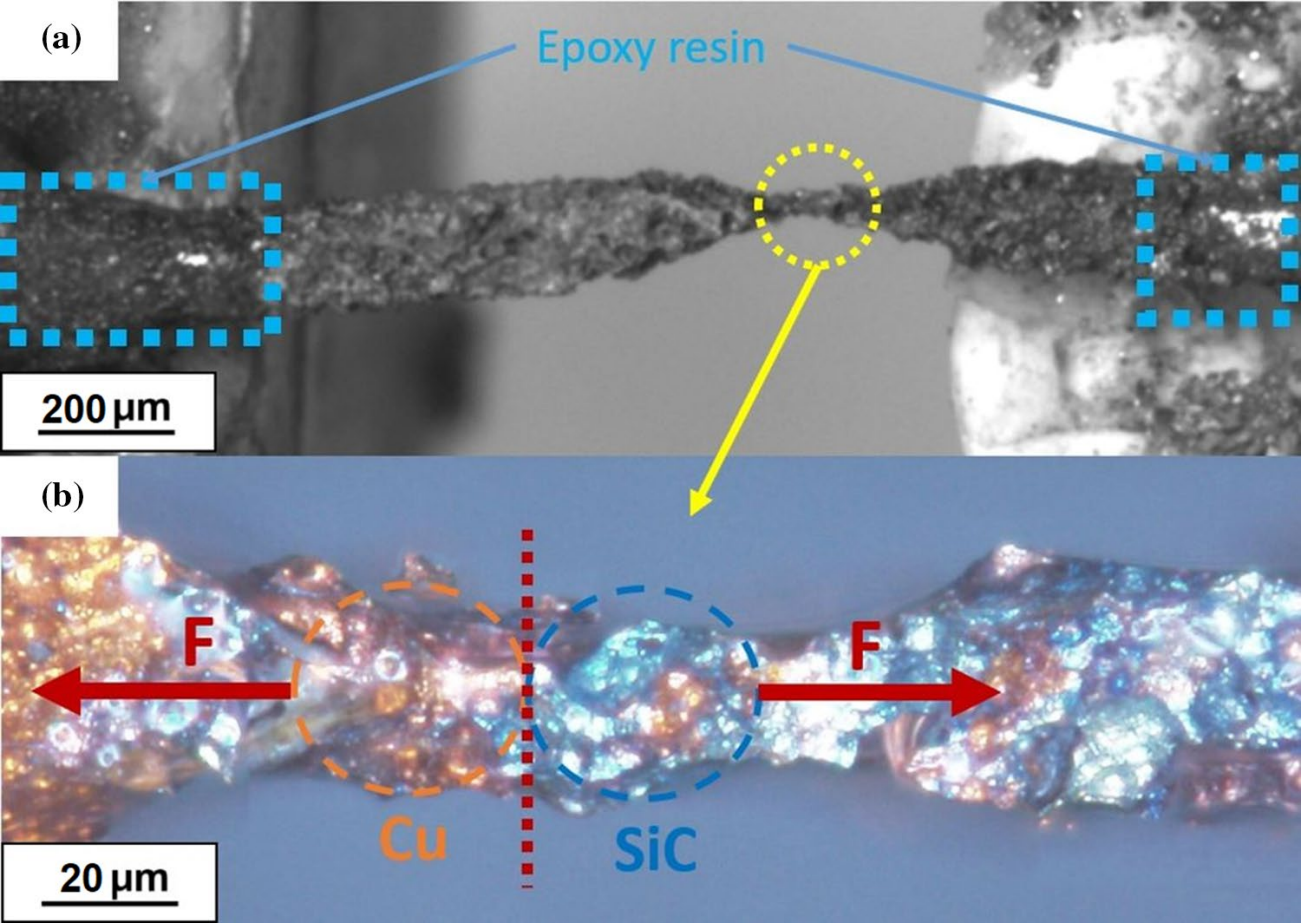
Cu–10%SiC composite manufactured by HPT **a** general overview by digital camera **b** Cu–SiC interface by optical microscope

At *the microscopic scale*, the microtensile strength test of Cu samples and the interfacial Cu–SiC bonding test has been performed. The microscopic tests were conducted via the procedure presented in Ref. [22]. Dog bone-shaped samples were prepared with cutting, a metal file, and grinding. Then, selective chemical etching was employed to further reduce a cross section to reveal the Cu–SiC interface. Cu samples were prepared with the same procedure and diameter was measured with an optical microscope. The composition of the etchant was: acetic acid (C_2_H_4_O_2_, 10 ml), hydrogen peroxide (H_2_O_2_, 10 ml), and distilled water (H_2_O, 80 ml). An example of a sample of Cu–SiC composite material in the form of a beam is shown in Fig. 4.

Samples were carefully mounted in tensile tester by means of epoxy resin to avoid standard mechanical squeezing (Fig. 4a) and etchant was applied precisely by pipette and was used to reveal a Cu–SiC interface and also to avoid destroying soft and thin sample while mounting (Fig. 4b). The displacement was forced by a stepper motor and the force was measured using a strain gage force sensor. The velocity of displacement was set to 0.01 mm/s. The ultimate tensile strength is defined as the force which breaks the rod at the interface, divided by the area of the interface:

1 $$s = \, {{F_{a} } \mathord{\left/ {\vphantom {{F_{a} } S}} \right. \kern-\nulldelimiterspace} S}$$

where *F*_*a*_ is the adhesion force between the metal matrix and a particle and *S* is the projected contact area. To determine the contact area, an optical microscope was used.

**Fig. 5:**
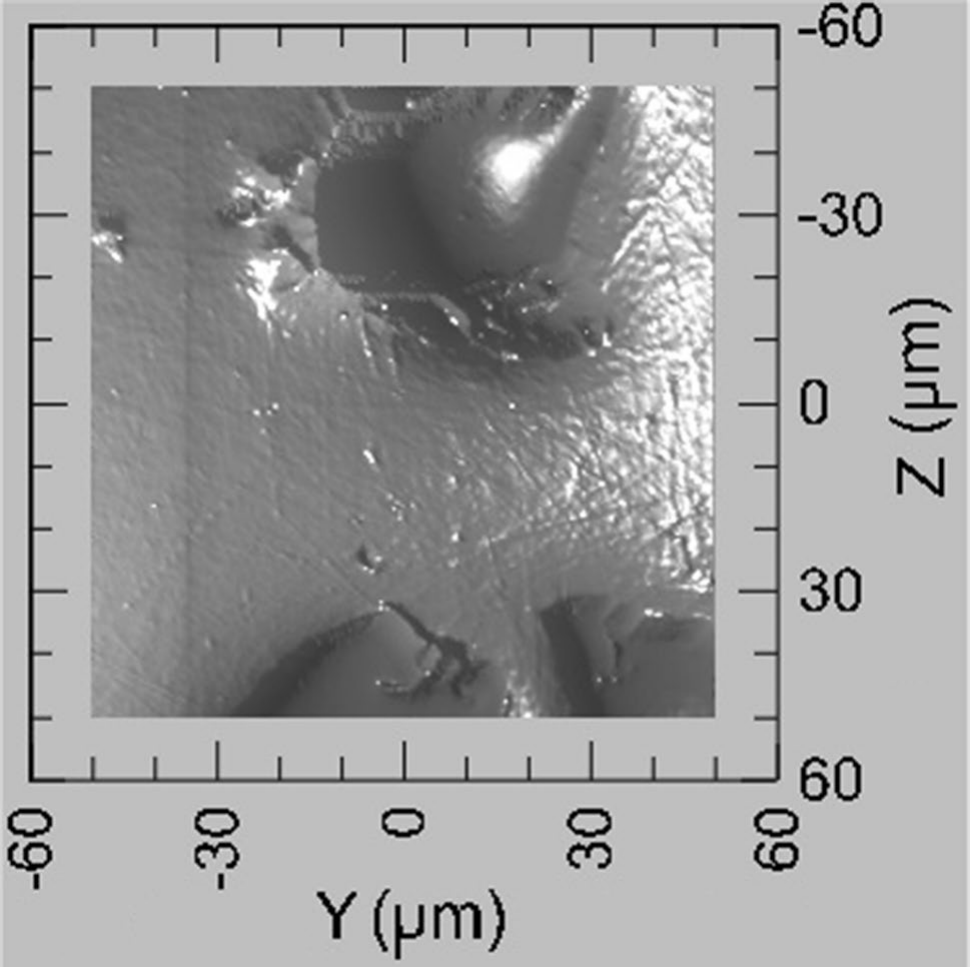
Example of the surface scan obtained by piezo-stage stage option

*At the nanoscale*, hardness, plastic and maximum depth were measured using the nano-indentation technique. Studies were performed on a NanoTest Vantage^®^ system developed by MicroMaterials Laboratory MML. All the experiments were conducted using a Berkovich-shaped diamond indenter. Before indenting each specimen series, the equipment was calibrated and the diamond area function (DAF) of the indenter tip was determined. A calibration procedure was completed by performing a series of indentations with a maximum load of 1 mN. As a test matrix with known properties, fused silica was used. The performed DAF calculations were in line with the ISO 14577 standard. Afterward, the calculated DAF was used at every stage of the analysis of the results. A thermal measurement period of 60 s was performed at the end of each indentation, the results of which are used to correct the indentation data during analysis. Several indentation techniques were employed:

➢ Targeted indentations: An area of 100 × 100 µm is scanned using the Berkovich indenter with a piezostage, providing a map of the surface (see Fig. 5) by moving over the surface and using a very low force (0.002 mN). However, because the imaging procedure is based on the mechanical contact of the sample and the indenter, and because the step size is 0.5 µm, the image resolution is lower than a typical AFM image. 12–15 indents using a maximum load of 1 mN, with 10 s loading, 5 s holding, 5 s unloading times are then performed in locations chosen after the mapping. The goal of this procedure was to indent intentionally: (i) metallic substrate, (ii) ceramic particles and (iii) interface-like region.
➢ Mappings: 10 × 10 indents were performed using a maximum load of 1 mN, with the following times: loading 10 s, holding 5 s, unloading 5 s; leaving 100 µm between indents to avoid with certainty any interaction between indents. Reported procedure allowed one to cover 1 mm^2^ of the surface and determine hardness profile over probed surface area.

After the indentations, all load–unload curves were fitted using the Oliver–Pharr method [23]. The goal of such detail analysis, involving conducting measurements in three volume scales was to perform a comprehensive structural and mechanical analysis of the studied system, obtain time- and scale-independent data which will help us to better understand occurring phenomena.

## Experimental results

### Microstructural characterization after HPT processing

**Fig. 6:**
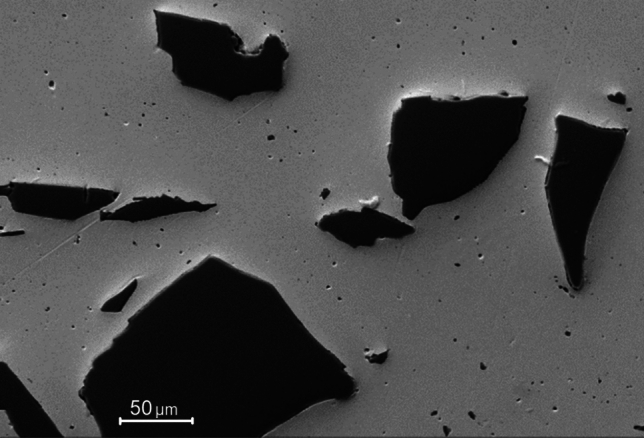
The representative SEM image of Cu–10%SiC composite sintered by SPS technique [16]

**Fig. 7:**
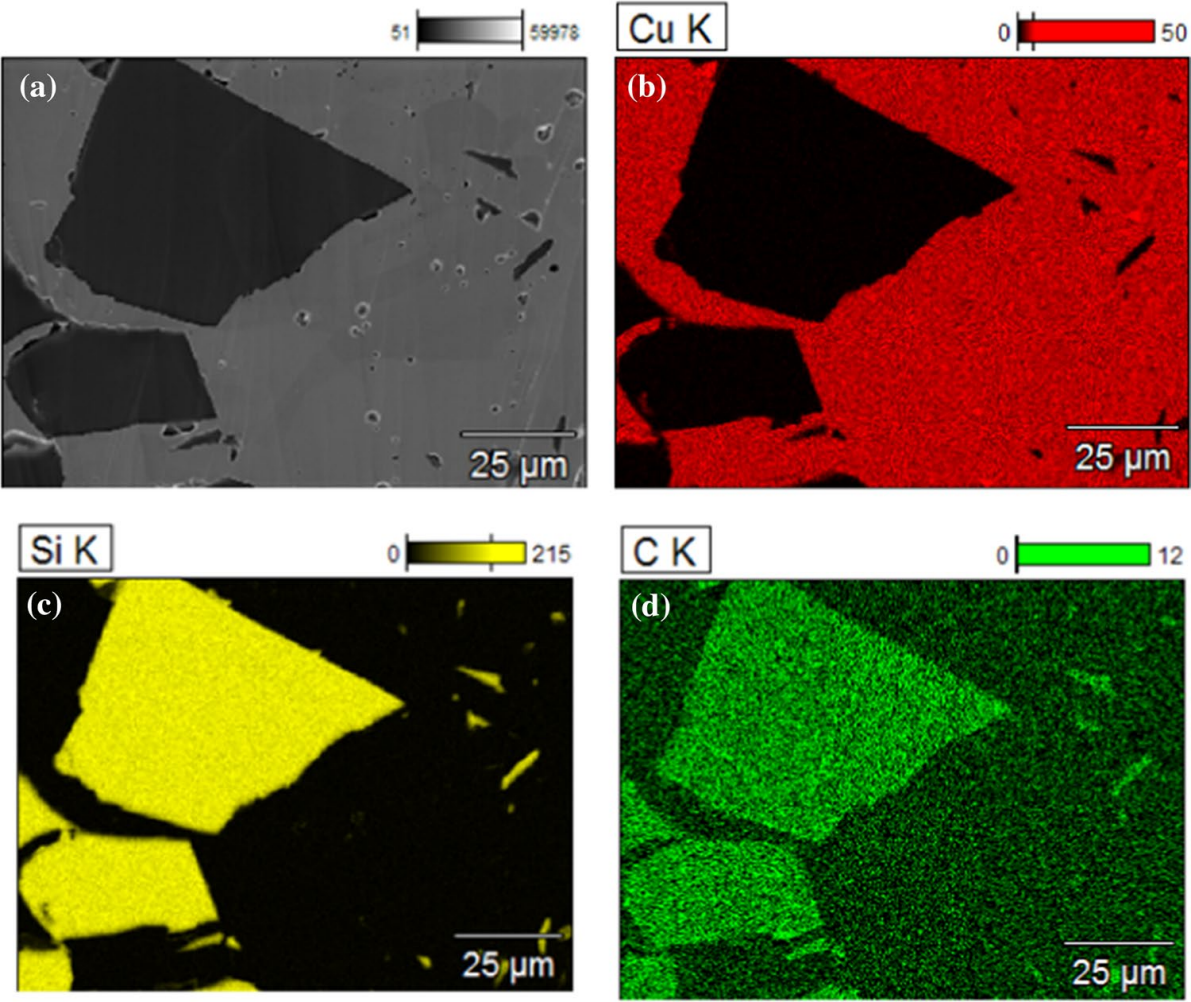
SEM image (**a**) and EDS maps of surface distribution of: (**b**) copper, (**c**) silicon and (**d**) carbon elements for Cu–10%SiC composite material sintered by SPS technique [16]

**Fig. 8:**
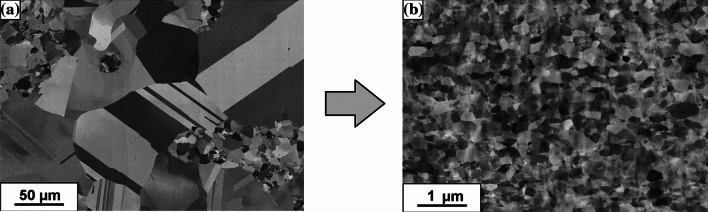
Evolution of copper microstructure after SPS (**a**) and subjected to the HPT process (**b**)

Processing by HPT leads to a significant change in the Cu and Cu–SiC composites. Based on microstructure analyses of SPS-processed samples performed earlier [15, 16], the Cu and Cu–SiC samples show grain inhomogeneity due mainly to the non-uniform rate of grain growth and recrystallization processes (Figs. 6 and 8a). The average grain size of a Cu matrix has been estimated at ~ 130 μm while ceramic particles have sizes from ~ 10 to ~ 100 μm. SEM imaging did not reveal any additional phases in the Cu–SiC composite structure which may be formed during the SPS processing. The distribution of chemical elements was investigated by EDS analysis, as shown in Fig. 7. On the other hand, the literature studies of Cu–SiC interphase brought a conclusion of formation of Cu_3_Si phase with a residual carbon layer due to the dissolution of silicon and carbon in the Cu matrix [15].

Finally, as shown in Figs. 6 and 7a, the microstructure of the SPS samples revealed a high fraction of nano- and micropores in the Cu matrix. The presence of residual porosity was confirmed by density measurements which gave a relative density of the Cu–SiC composite fabricated by SPS at ~ 98% [16]. The measured density of copper samples after SPS was 8.85 g/cm^3^ (relative density 99.3%).

These microstructural characteristics may affect the material strength in an undesirable way but it appears that their influence is minimized by the use of HPT processing. Due to the application of significant compressive and shear stresses during the plastic deformation, practically no pores or voids were visible in the metal matrix (Fig. 8b). This effect is confirmed by density measurements for the Cu–10% SiC and Cu–20% SiC composites processed by HPT where the measured values were ~ 99.5% ± 0.2%. In the case of copper samples, the density changed slightly to the value of 99.8%*.* Furthermore, the average Cu grain size after HPT processing was ~ 350 nm which is significantly smaller than for the SPS sample [13].

**Fig. 9:**
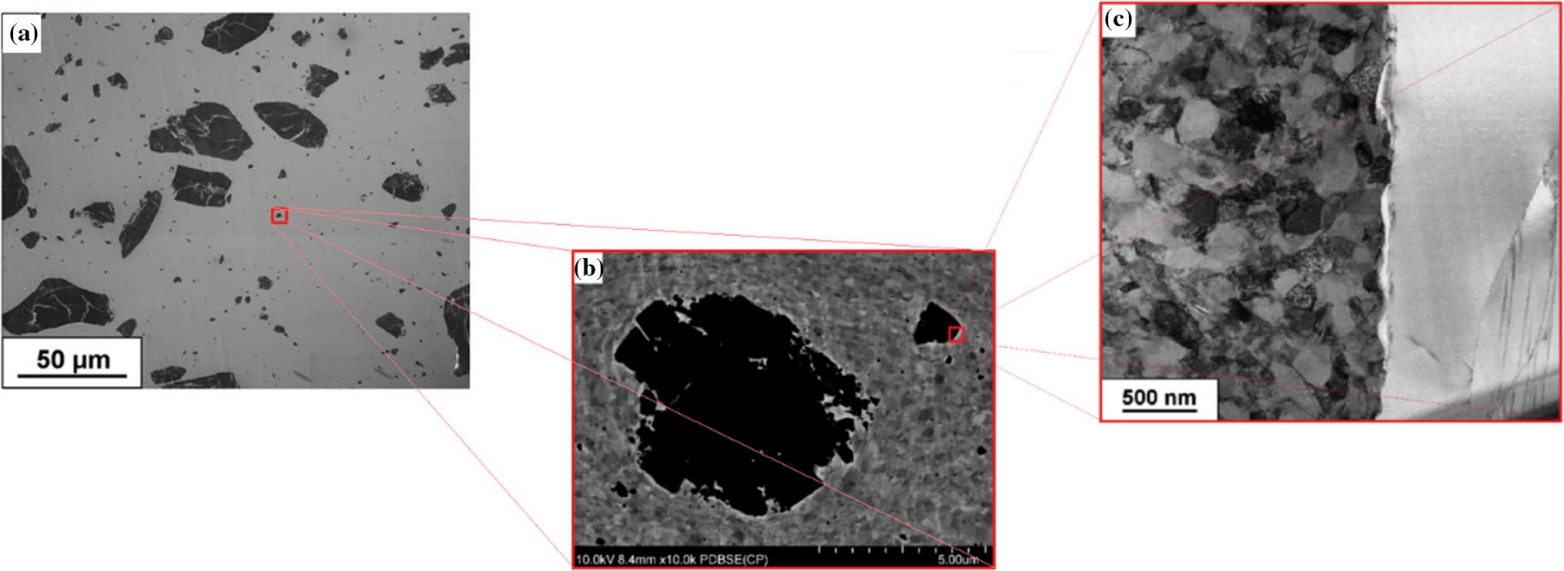
The microstructure of Cu–20%SiC composite after HPT process (**a**, **b**) with additional TEM images (**c**)

On the other hand, the impact of large stresses during HPT was not sufficient to fully fragment the largest SiC particles and these are visible in the samples where they tend to display numerous internal cracks and discontinuities (Fig. 9). In practice, many of the large ceramic particles become fragmented into smaller particles thereby creating agglomerates that reduce the overall strength of the system.

Moreover, due to their low fracture toughness, most of the ceramic SiC particles evolve into clusters of particles having sizes between ~ 0.05 and ~ 6.0 µm. The microstructure also displays a high fraction of fine SiC particles with sizes of ~ 50–700 nm, which are dispersed and reasonably homogeneously distributed within the Cu matrix. Significant changes of metal–matrix and ceramic reinforcement structure should be reflected in the mechanical behavior of the material at three investigated scales.

### Mechanical properties at the *macroscopic* scale

**Fig. 10:**
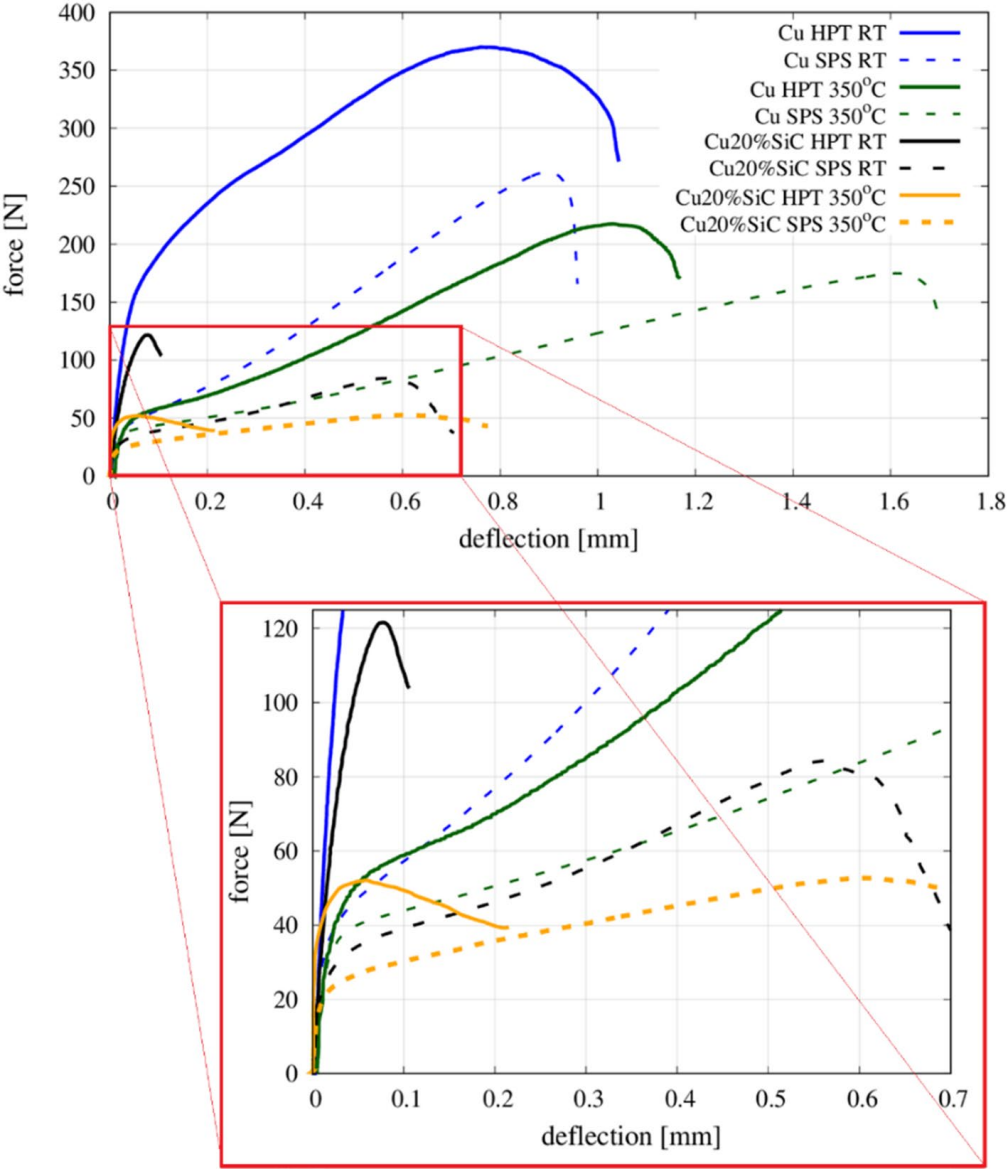
Representative force–deflection curves of copper and Cu–20%SiC composite processed by SPS and HPT registered during SPT test in RT and 350 °C

**Fig. 11:**
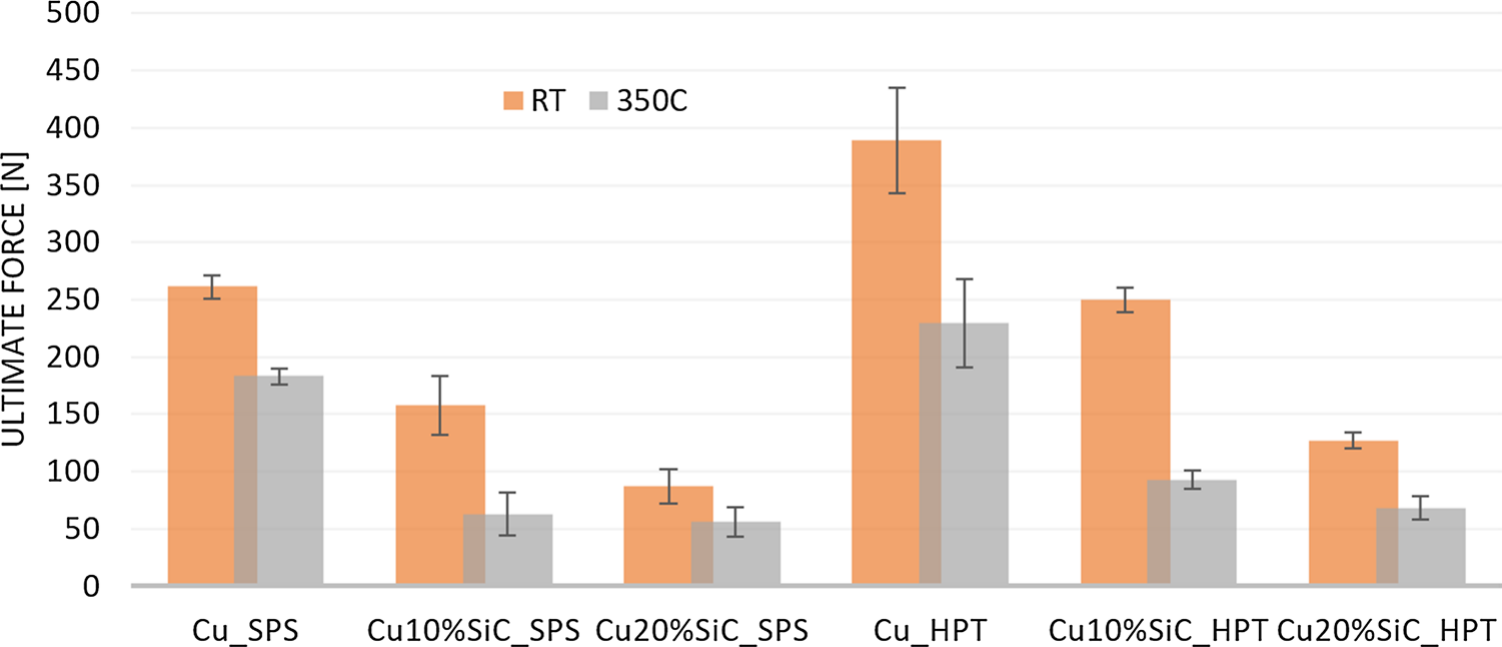
The ultimate force results of the small punch test performed at the macroscopic scale

Revealed microstructure evolution after HPT processing should be reflected in the adequate evolution of material’s mechanical properties at the macroscopic scale. Here, the mechanical properties were evaluated by small punch tests on Cu samples and Cu matrix composites with 10 and 20% vol. content of SiC samples fabricated by SPS and HPT. Representative force–deflection (F–D) curves of Cu and Cu–20%SiC samples registered during the SPT test in RT and 350 °C have been shown in Fig. 10. Based on F–D curves, the ultimate force has been evaluated and presented in Fig. 11.

It is readily apparent that the *F*_u_ was higher for all specimens after plastic deformation processing by HPT, where the increases were of the order of 64–70% for both Cu–SiC composites tested at RT and 350 °C, but it was associated with a loss of plasticity, measured by specimen deflection to failure. Loss of plasticity of HPT samples is especially evident in the case of composites, which indicates up to five–six times lower deflection in respect to the SPS one. In the case of Cu samples, this effect is much lower both at RT and at a higher temperature. Furthermore, the impact of temperature applied during tests on material's mechanical properties is apparent. Similar to the case reported in Ref. [24], an unfavorable influence of temperature on Cu and composite strength during small punch testing was shown. On the other hand, even though all materials deformed at 350 °C revealed smaller ultimate force, they show a higher deflection to failure in comparison to RT. Finally, the effect of the SiC reinforcement on the Cu matrix was studied for both the SPS and HPT specimens and, as expected, for SPT the highest maximum force was obtained for Cu at both studied temperatures.

**Fig. 12:**
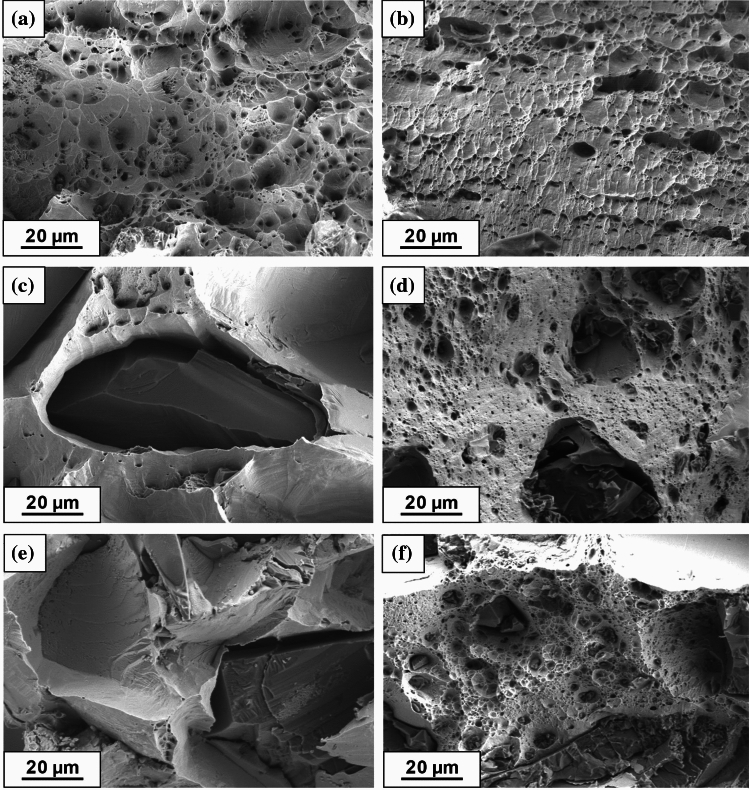
Representative images of the fracture surface after test: **a** Cu SPS, **b** Cu HPT, **c** Cu–10%SiC SPS, **d** Cu–10%SiC HPT, **e** Cu–20%SiC SPS, **f** Cu–20%SiC HPT

The evolution of the material's mechanical properties is accompanied by a shift in damage mode. Figure 12 shows representative images of fracture surfaces of the samples after macroscopic strength tests. Both Cu samples fabricated by SPS and HPT (Fig. 12a, b) exhibit ductile character with evidence for dimples, micro-voids and necking in macro observations. It is worth noticing that the mentioned features are more apparent in the SPS sample case.

The Cu–10% SiC and Cu–20% SiC SPS samples displayed similar fracture surfaces, as shown in Fig. 12c, e. Cracks were initiated at micro-voids and discontinuities occurring at the interfaces between the SiC particles and the Cu matrix and propagated intergranular along with the weak bonding without damaging the particles. Conversely, HPT composite sample fracture indicates the combination of trans- and intergranular damage as shown in Fig. 12d, f. On the one hand, a ductile fracture can be seen within the Cu matrix with an additional cleavage fracture of the SiC particles, and on the other hand, the ceramic reinforcement is pulled out from the metal matrix, pointing to intergranular damage along the interface zone.

### Mechanical properties at the *microscopic* scale

The main aim of the microscopic experiments is to qualitatively determine the tensile properties of Cu and the interface between the matrix and ceramic SiC reinforcement in the context of mechanical improvement of each component of the composite after HPT. The tensile experiments have been performed using the procedure presented in Sect. 2, taking special care to prepare samples.

**Fig. 13:**
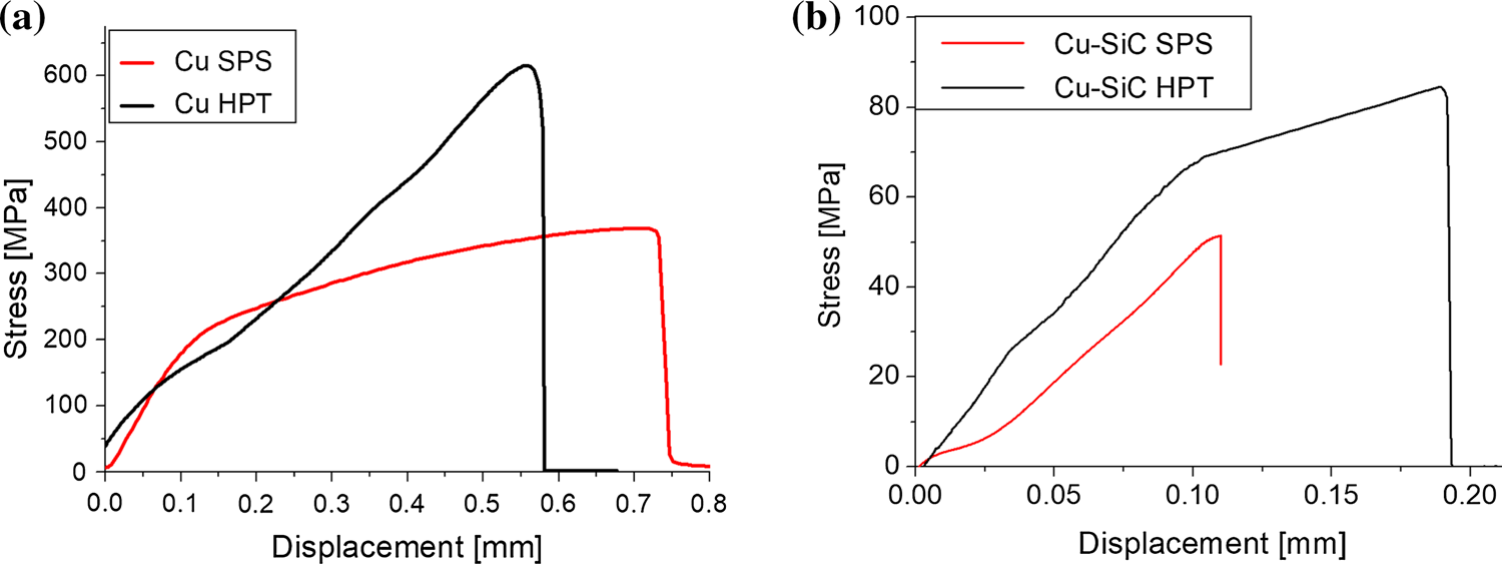
Engineering stress–displacement curves of microtensile testing for **a** Cu–Cu, and **b** Cu–SiC bonding

Since the velocity of the holder and the cross-sectional area of the broken bonding are known, it was then possible to determine the engineering stress-displacement curves. It would also be interesting to evaluate strain (however, the estimation of the initial length or the length of the reduced section is hindered). Nevertheless, representative curves of the tensile testing are shown in Fig. 13a, b for Cu and Cu–10% SiC, respectively, in the as-sintered and the as-deformed stage. The results of Cu bonding fabricated by SPS brought a weaker response than HPT. Furthermore, Cu SPS exhibited significant plastic deformation, whereas Cu HPT was more brittle with a dominant elastic range. In the case of interfacial bonding tensile strength test of Cu and SiC particles, both SPS and HPT samples indicate a pure elastic and brittle character in the full range of deformation. The samples broke in a brittle manner. However, for the second one, the change in a curve slope is visible. We tentatively attribute it to the change in the resultant sample stiffness due to the delamination of interfaces other than the studied particles. It is worth mentioning that during epoxy resin solidification, tensile force increased from 0 to 4 N (about 8% of max. tensile force). To minimize such an effect, one should move a holder to obtain force equal to 0 N a couple of times during solidification. As the time and velocity of the holder are known, it is possible to determine displacement and knowing the area of the initial cross-section one can calculate engineering stress so force–time and engineering stress–displacement curves are of the same shape.

**Fig. 14:**
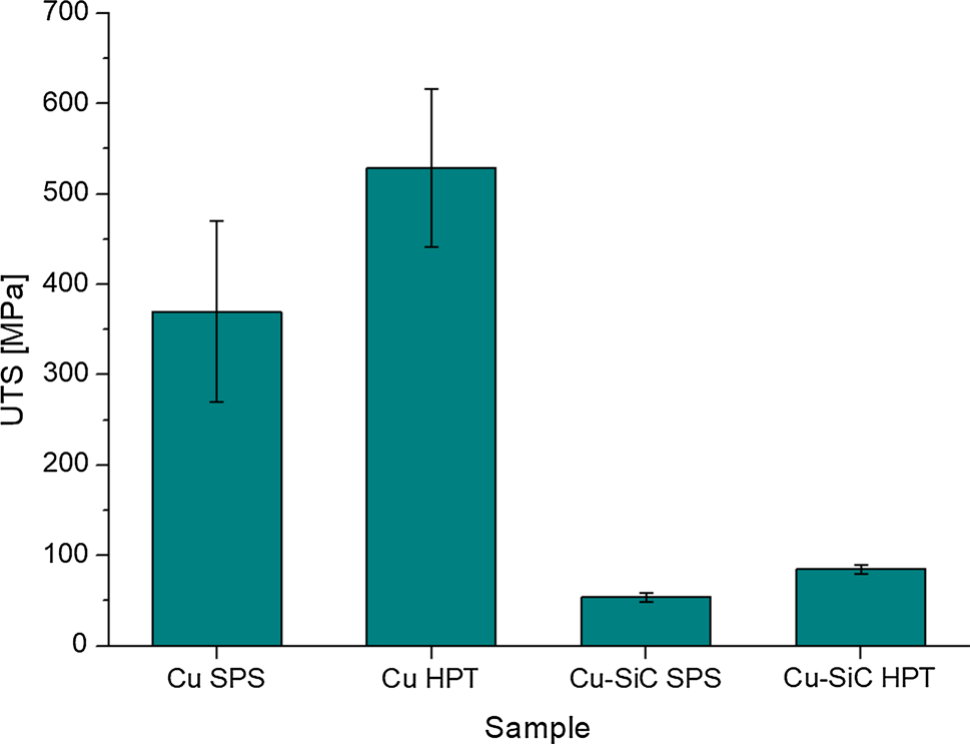
Results of microtensile strength measurements

The values of the ultimate tensile strength UTS, shown in Table 1 and Fig. 14, demonstrate significant material enhancement after the HPT processing for both pure Cu and Cu–SiC bonding. The ultimate tensile strength of Cu samples increases by around 40%—from 370 to 529 MPa. The mechanical enhancement of Cu–SiC bonding is more spectacular than the Cu matrix—the rise of UTS is by around 60% from 54 to 85 MPa.

**Table 1:** Results of micro-tensile strength measurements

	Cu SPS	Cu HPT	Cu–SiC SPS	Cu–SiC HPT
Ultimate tensile strength [MPa] with standard deviation	**370** ± 100	**529** ± 87	**54** ± 5	**85** ± 5

The micro-tensile testing reveals the advantageous impact of HPT on the micromechanical component of the composites, however, several unfavorable aspects of the micro-tests ought to be mentioned. Uncertainty of measurements can be caused by a couple of factors: the rapture of the sample takes place not necessarily in the thinnest cross section and also could occur in the plane non-perpendicular to a direction of tensile force, mounting samples with epoxy resin does not provide precise control of collinearity of sample axis and tensile force direction, machining of the samples could interfere in internal stresses. Moreover, the real contact area is estimated as the area of cross section before the tensile test. Values of uncertainty are calculated as expanded uncertainty of combined standard uncertainty which takes into account the factors mentioned above.

### Mechanical properties at the *nanoscale*

**Fig. 15:**
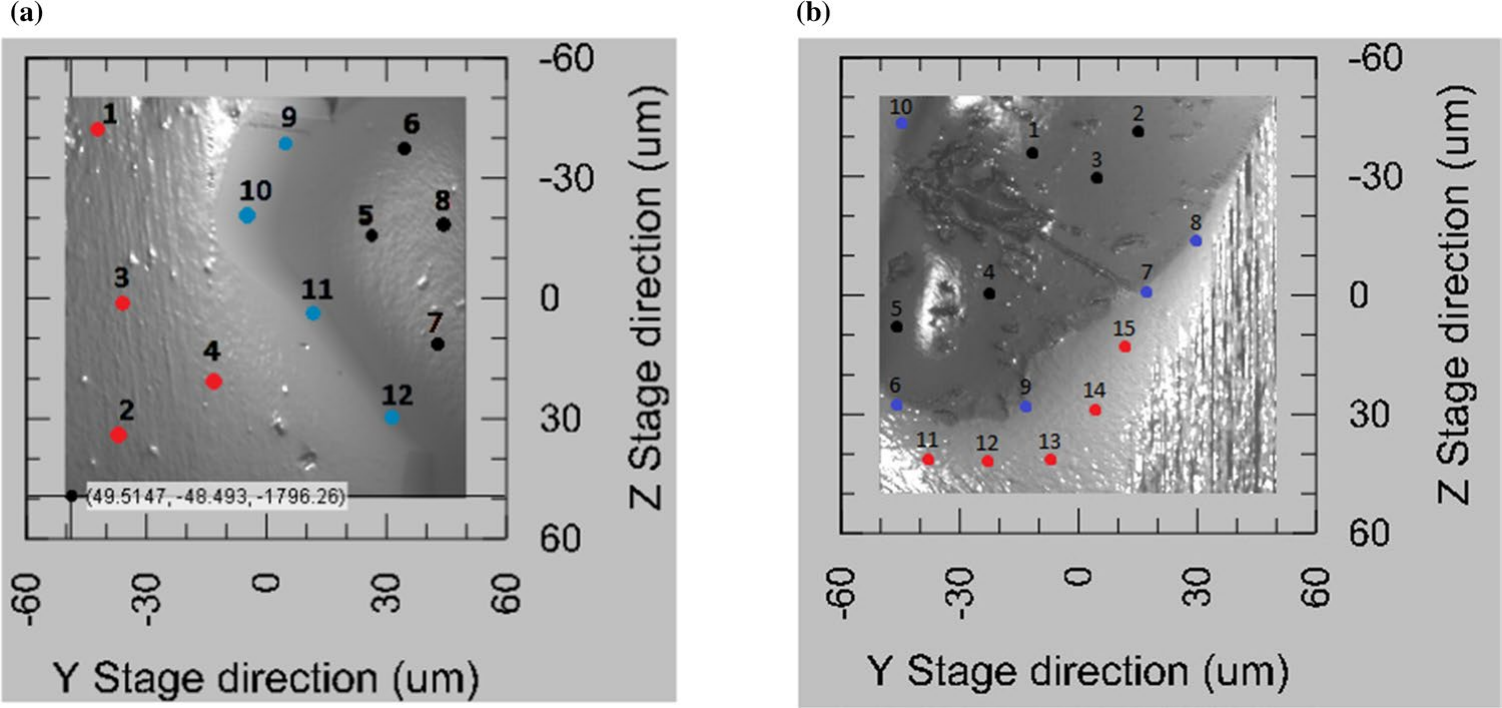
Surface scan performed using piezostage option. Marked red dots depict indentation made on a metallic substrate, black at ceramic particle and blue at interface-like region. L–D curves are presented in Fig. 13: **a** Cu–20%SiC SPS and **b** Cu–20%SiC HPT samples

**Fig. 16:**
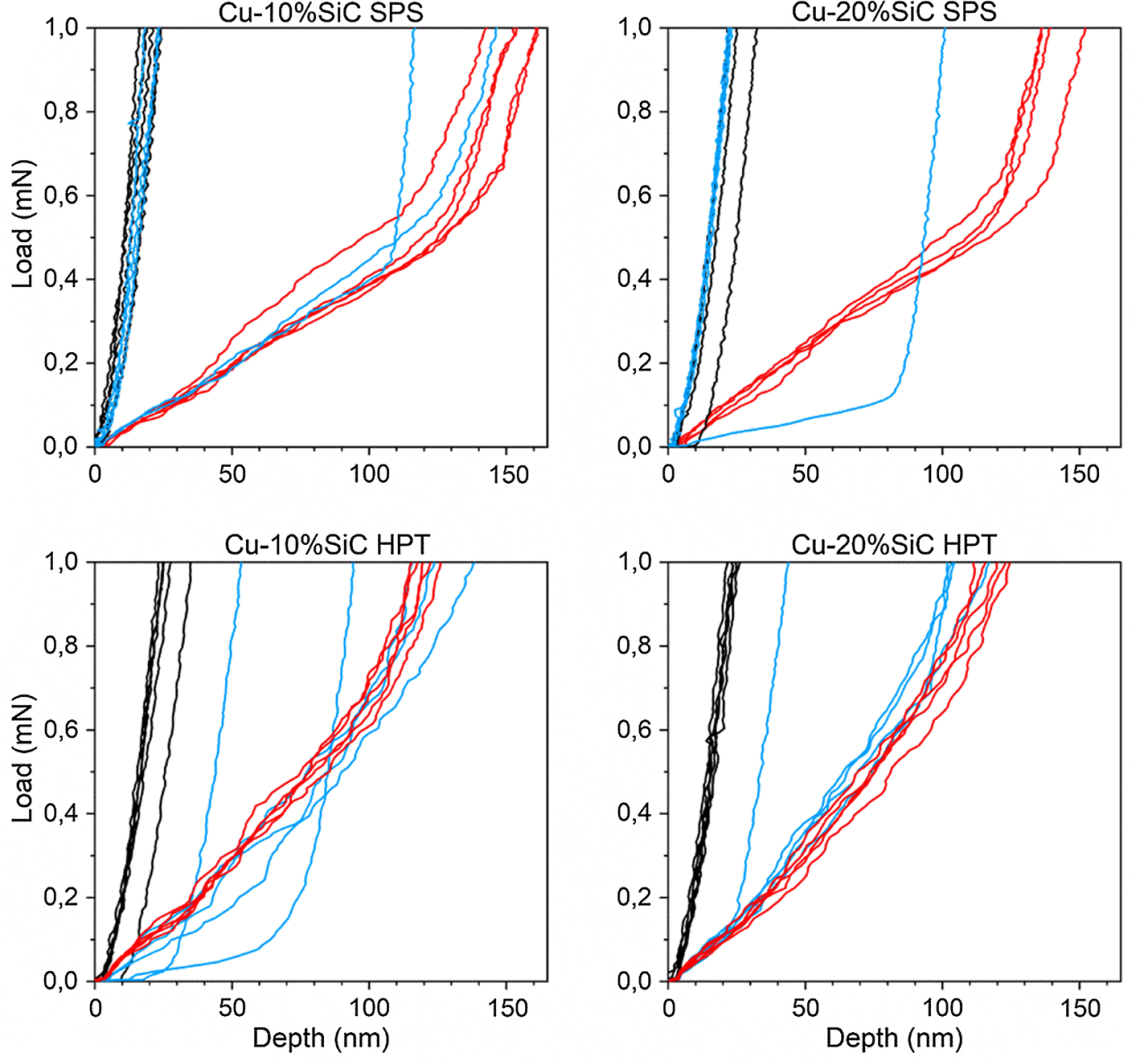
Individual load–displacement curves obtained from targeted indentations

**Fig. 17:**
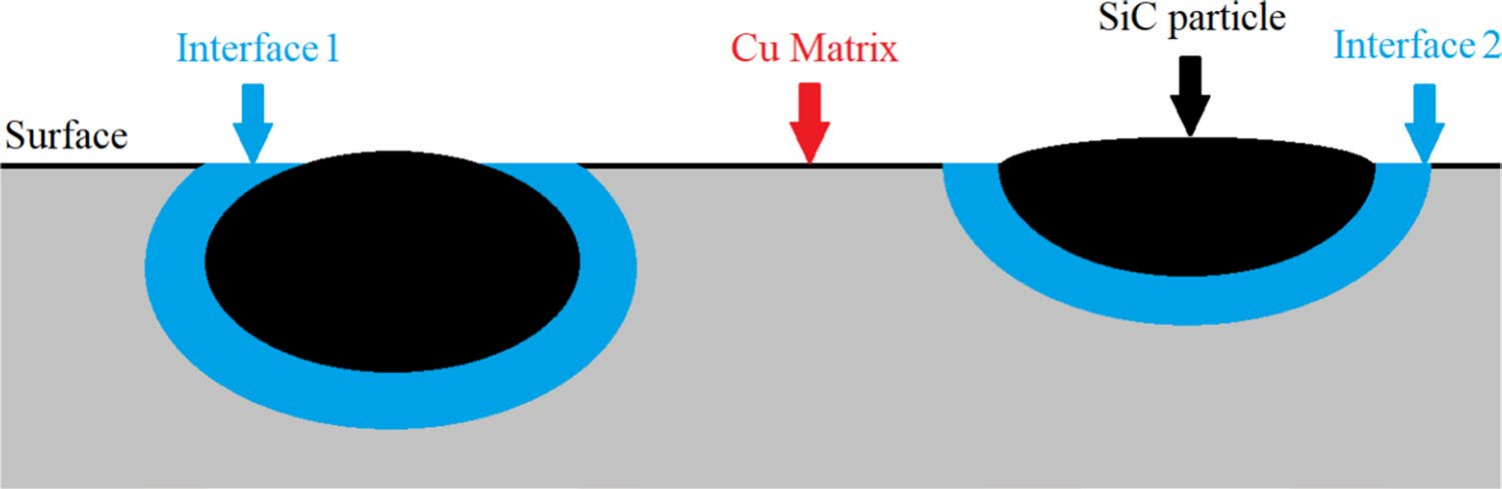
Schematic representation of a cross section of the bulk of Cu–SiC, near the surface, along with examples of indentation points

The nano-mechanical properties of the ceramic phase, the metallic matrix and their interface region were measured via nano-indentation technique using two independent procedures: targeted indentation of selected areas and defined surface mapping. Targeted indentations were performed to collect data from all three phases. Figure 15 displays examples of the Cu–SiC composite regions, with sizes of 100 × 100 µm^2^, submitted to targeted nano-indentation campaign. Dots marked on the images indicate positions of the nano-indentations. To differentiate between probed phases, individual colors were assigned. For example, the black color depicts indentation made at the ceramic phase, red marks the metal phase and blue stands for probing the interface region. These colors are used in Figs. 15, 16 and 17. One should explain that the images presented in Fig. 15 are made via mechanical contact of the indenter tip with the sample surface using the so-called reverse AFM procedure. Details of this methodology can be found in Ref. [19].

The nano-indentation load–displacement curves showed in Fig. 16 are a result of targeted indentations (no data smoothing was made). All nano-indentations were made under 1 mN load which corresponds to the penetration depth of ~ 20–160 nm, depending on the probed phase. One should explain that a relatively small indentation force was used in the targeted indentation campaign to accurately compare different regions of the composite by probing its volume with the same load. Since SiC is a hard material that is embedded in a soft Cu substrate, one may expect that application of high load, applied to SiC particle may result in its rotation or partial displacement in the softer Cu. Therefore, to obtain an accurate estimation of the nano-mechanical properties of each phase, targeted indentations were made at least four times and the average values are given in Table 2.

**Table 2:** Hardness, plastic and maximum depth recorded during nano-indentation test of individual phases: Cu—copper metallic matrix, I—interface region and SiC—ceramic silicon carbide reinforcement

Material	Hardness (GPa)			Plastic depth (nm)			Maximum depth (nm)		
	Cu	I	SiC	Cu	I	SiC	Cu	I	SiC
Cu SPS	1.1	–	–	150	–	–	156	–	–
Cu HPT	1.5	–	–	125	–	–	132	–	–
Cu–10%SiC SPS	1	17	31	154	60	12	160	68	21
Cu–10%SiC HPT	1.6	2.7	20	118	100	18	124	108	28
Cu–20%SiC SPS	1.2	19	31	138	34	12	144	42	21
Cu–20%SiC HPT	1.6	3.3	25	117	90	15	123	97	24

The authors would like to point out that indentation data describing interface results, in this case, should be treated only as additional information showing a general tendency of the material/phase evolution for the following reasons. First, as schematically shown in Fig. 17, depending on the orientation and position of the SiC embedded in the Cu substrate, the interface zone may not be placed directly beneath the indent. For example, the SiC may be placed at a short distance beneath the indent (case “interface 1”), or be further from the indent position as the depth increases (case “interface 2”). Both cases result in deterioration of the plastic deformation triggered by indentations, which impacts obtained results.

Although in both cases, the indents would be placed at similar distances from the boundary of the SiC and Cu substrate (as seen from the surface), they would yield considerably different results. Actually, it has been agreed that indenter probes volume of the material extending approximately even 10 times the indentation depth along with the force application, and perpendicular to that force approximately 6 times the indentation radius, near the surface. All these result in the formation of the half-ellipse. This means that invisible objects below the surface may affect the mechanical properties evaluated from the load–displacement curves. Second, the resolution of the image obtained for targeted indentation is not as good as with AFM, for example. In Fig. 15a, the borders/endings of the SiC particles are not obvious, hence, when choosing indentation coordinates, one cannot be sure whether indeed indentation is made close to the SiC, or directly above it. Third, because of the large difference in mechanical properties between the matrix and ceramic reinforcement, the SiC particles tend to protrude after polishing, making the surface uneven and causing automatic surface detection at the interface, which increases inaccuracy. Lastly, indents may not be performed exactly where the coordinates are placed, which is relevant in the case of precise indentation at a narrow interface zone. For these reasons, the load–displacement curves showed in Fig. 16, obtained from targeted indentations of interface areas, are not perfectly uniform. However, on the other hand, they should not be significantly burdened with the abovementioned effect. Despite these controversies, the nano-indentation technique is currently the only method that allows for probing small volumes of the materials. Since the goal of this work is to understand and explain the mechanical behavior of a very challenging system like Cu–SiC, we took the effort and despite some procedural pitfalls, we intend to critically assess obtained nano-mechanical data, as this may have critical importance in further development of this material and understanding its mechanical properties at larger scales.

**Fig. 18:**
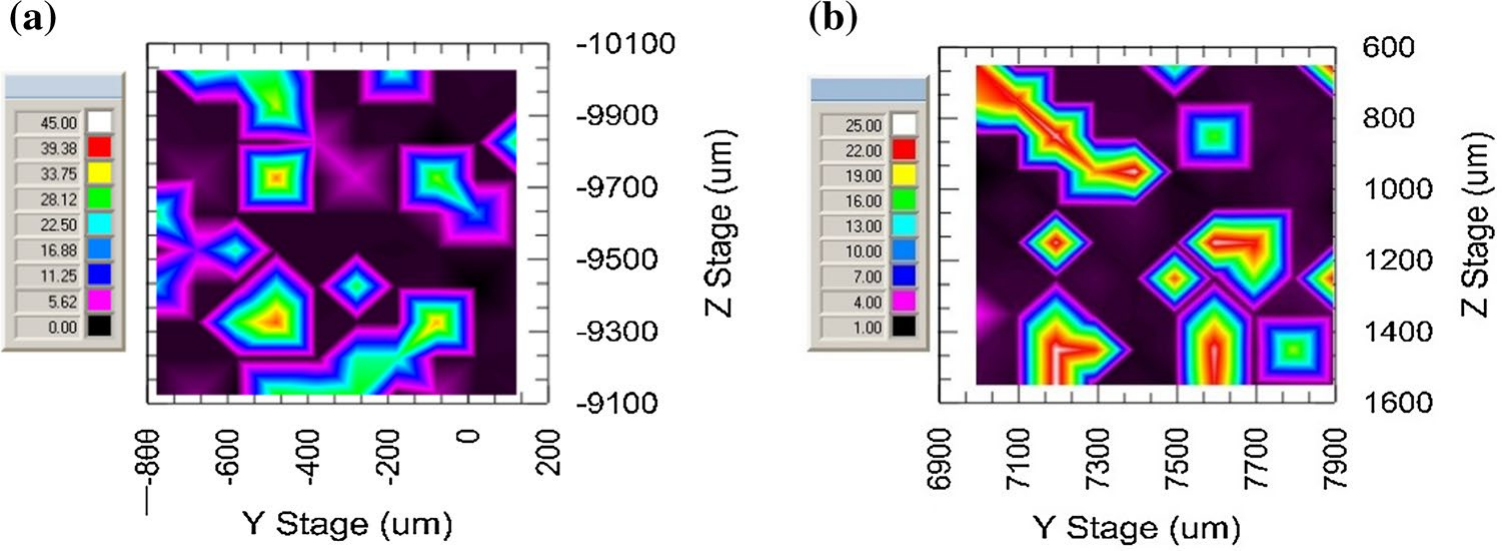
Mapping of hardness results for: **a** Cu–20%SiC SPS and **b** Cu–20%SiC HPT samples. Hardness values in GPa

**Fig. 19:**
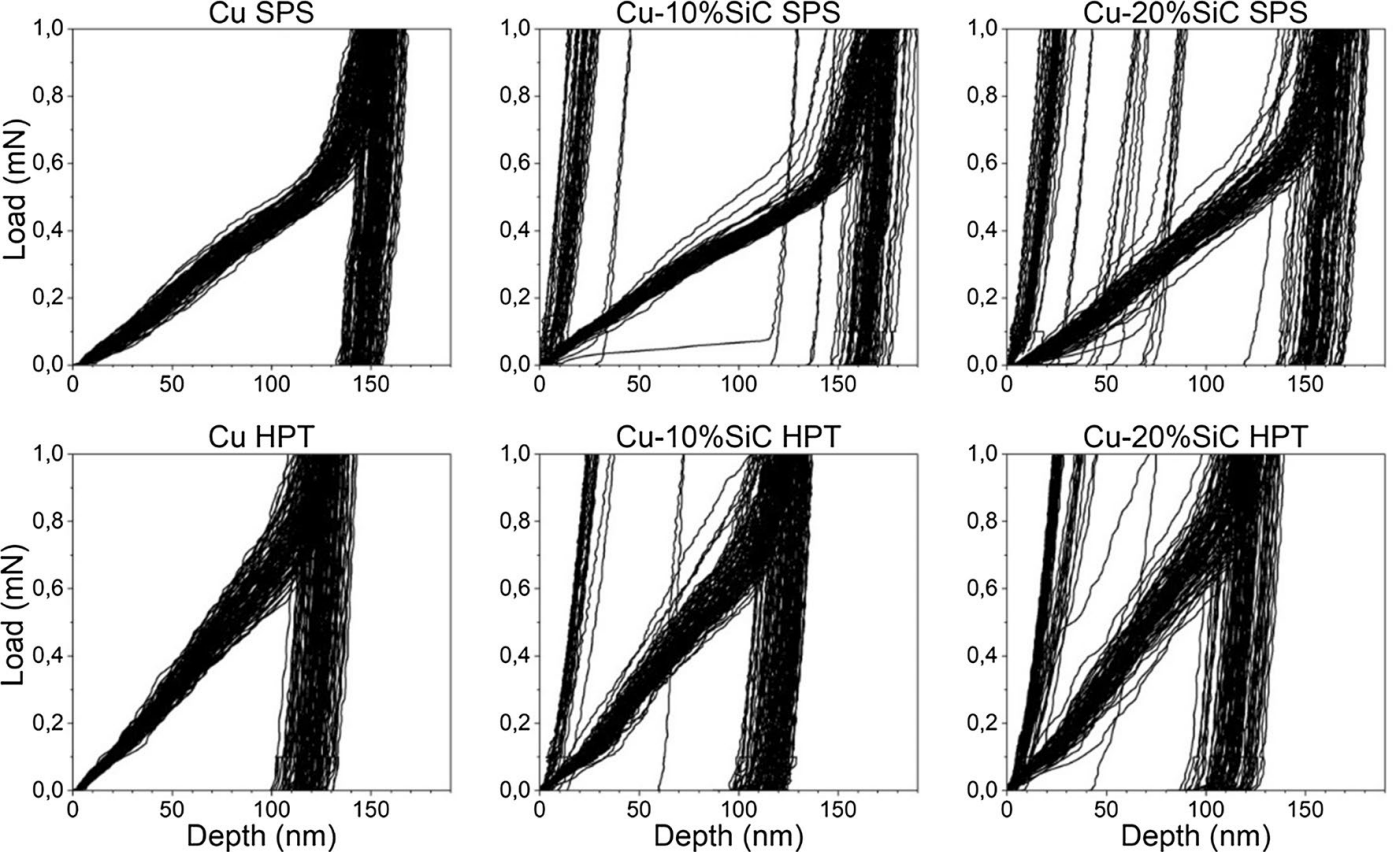
Load–displacement curves obtained from mapping tests recorded on the specimens manufactured via SPS and HPT techniques, with different SiC additions

Following the targeted indentation, mapping experiments were performed. Results of these tests are presented in Figs. 18 and 19. The presence of SiC can be easily distinguished from the matrix in both figures, as they are characterized by much higher H values. As mentioned previously, the indenter probes volume of the material which extends further than the indent itself. In our investigation, the information is collected from the volume extending approx. 1.5 µm in depth in the case of Cu SPS, and approx. 1.3 µm in the case of Cu HPT. Recorded nano-mechanical data show that specimen produced by HPT has a higher hardness than that produced by the SPS technique.

However, this hardness might be overestimated due to the different plastic behavior recorded at a different scale. Under micro-indentation (with loads up to 5 N, i.e., 5000 times greater than with nano-indentation), Cu HPT shows more pile-up around the indenter than Cu SPS, leading the device to underestimate the indentation depth, hence overestimation of the hardness. Further analysis would be required to verify whether this is also the case with the loads employed during nano-indentation (using AFM, or analysis of indent cross-sections in SEM, for example).

Finally, one may notice different scales between Fig. 18a, b. This is due to the fact that in this particular case, we probed an area with a much bigger amount of SiC particles. Also, one cannot exclude the possibility that below the Cu substrate, large SiC grain is present. All this will naturally increase the recorded nano-mechanical properties of the system. Alignment of the scales will hide the true disproportion between soft and hard parts of the studied system.

## Discussion

Generally, one main conclusion can be made from presented mechanical tests performed on three separate scales. As was expected, the high-pressure torsion has affected significantly both Cu samples and Cu–SiC composites. Explanation of such enhancement of metal–matrix composite has its origin in the strengthening effects of the material across the scales at each composite component: metal matrix, ceramic reinforcement and metal–ceramic interface.

### The impact of HPT processing on the metal matrix

The application of HPT refers to substantial improvement in the Cu mechanical properties. In practice, obtained grain refinement and elimination of residual porosity (Fig. 8) result in the increase of the strength and hardness through the Hall–Petch relationship but there is also a slight reduction in the elongations to failure as determined in micro-tensile tests (Fig. 13). This effect is also observed at the nanoscale in Fig. 16. Nano-indentation tests show an increase in hardness and a decrease in plastic depth in the Cu matrix after HPT as documented in Table 2. This effect is also visible in the various load–displacement curves obtained from targeted indentations which differ depending on the processing method as shown in Fig. 16. Thus, the curves of the Cu matrix from the SPS samples in Fig. 16a, b display a relatively substantial elastic range and subsequently the plastic one, while those after HPT are steeper without a clear plastic zone [25].

The nano-indentation study offers another conclusion related to the HPT materials. Specifically, the presence of SiC particles leads to an increase in hardness of the Cu matrix compared to Cu samples. As was confirmed earlier at the micro-scale during micro-indentation tests [13], the hard and tightly packed SiC particles obstruct the movement of dislocations.

Presented results are generally consistent with the behavior of ultrafine-grained and/or nanocrystalline FCC materials [26]. The main feature of these materials, including Cu HPT and Cu matrix composite HPT, refers to an induced large number of various types of defects: a high density of dislocations, the significant volume fraction of grain boundaries [27]. As a result, the favorable combination of high strength and satisfied ductility can be preserved [28].

### The impact of HPT processing on the ceramic reinforcement

The mechanical and microstructural properties of SiC particles change dramatically due to the application of HPT processing. Several reasons explain such a transition. Generally, the particles in SPS samples retain their initial dimensions within the range of ~ 10 to ~ 100 µm despite the mixing process, which has little or no effect on the ceramic particle size [13]. The bonding between the SiC reinforcement particles does not display a cohesive/diffusive character because of the limited sintering processing conditions. Consequently, small amounts of microporosity are produced between the ceramic particles and this is a potential source for crack initiation where the HPT processing improves the homogenization of the composite microstructure and produces a more uniform distribution of SiC particles. For the HPT Cu–20% SiC samples, this leads to significant refinement of ceramic particles (Figs. 9 and 12d, f).

Ceramic nanoparticles, obtained as a result of HPT processing, create an advantageous system with reference to the macroscopic composite strength and they provide a high mechanical resistance due to several strengthening mechanisms such as the Orowan effect or the load-bearing effect or load transfer between the matrix and the reinforcement [29, 30]. This reinforcing mechanism is effective only if a strong cohesion is achieved between the matrix and the reinforcement [31].

A well-defined dual impact of HPT processing on the mechanical properties is evident in the nano-indentation investigation. First, there is a weakening of SiC after HPT with respect to SPS for all compositions. The weakening resulting in the lower hardness of the ceramic phase of the HPT samples is attributed more to the cracking and damage of the SiC particles after application of sizable forces during processing than the plastic deformation resistance. In contrast to the ceramic particles after HPT, the solid and undamaged SiC reinforcement after SPS shows higher resistance to the applied load during nano-indentation testing.

Second, after HPT, the SiC particles exhibit higher hardness levels as the ceramic content rises. Thus, applying HPT to Cu–20% SiC composites leads to more effective fragmentation and in this way, it lowers the numbers of relatively large SiC particles while simultaneously increasing the numbers of nanoparticles, which benefits the mechanical properties at the nanoscale.

### The impact of HPT processing on the metal-ceramic interface

The presence of SiC particles increases the hardness of the Cu matrix due to the Orowan strengthening mechanism but, at the macroscopic scale, the Cu–SiC composites fabricated by SPS and HPT both exhibit lower strengths compared to Cu samples (Figs. 10 and 11). In practice, the addition of ceramic particles to the metallic matrix leads to an increase in the number of metal–ceramic interfaces and these are the weakest links when measuring the material strength [24]. These interfaces are defined as two-dimensional zones wherein one or more material parameter exhibits a discontinuity [32] and this has a significant impact on the composite properties, such as fracture behavior [33], the toughness [34] or/and wear behavior [35]. The importance of the interface may be examined in terms of two general aspects. First, the physical state of the interface and the presence of material defects that may change the properties at or near the interface itself. Second, the chemical state of the interface since a new phase may form during processing due to interactions between the metal–matrix and the ceramic reinforcements.

These effects are found in Cu–SiC composites processed by SPS in which the physical structure of the Cu–SiC interface reveals a high fraction of nano- and micropores. Furthermore, new phases may be formed in the matrix-reinforcement bonding region which will influence the mechanical properties [15]. In the area of the interface zone, SiC decomposes to silicon and carbon in contact with Cu and the Si dissolves into the Cu matrix to form a Cu_3_Si phase with a residual carbon layer [36, 37].

In practice, the microstructural features of the Cu–SiC interface zones of samples prepared by SPS contribute to the mechanical response. Assuming that the composite strength at the macroscopic level is mostly affected by the metal–ceramic interface, experimental results obtained at the microscopic level should assist in interpreting the data. However, there is no evidence for any voids, de-bonding or cracking in the contact zones between the Cu matrix and the SiC reinforcements after HPT (Fig. 9). Processing also improves the adhesion between the soft matrix and the hard particles through an enhanced dislocation density strengthening mechanism [38]. The metal–ceramic interfaces become areas of residual plastic strain and this increases the dislocation density due to the matrix/reinforcement elastic modulus mismatch and work hardening during the deformation process [39]. Additionally, the high compressive and shear stresses lead to large microstructure transitions within the Cu–SiC contact zones, leading to the annihilation of Cu_3_Si and the residual carbon layers. This means that the HPT-processed Cu–SiC composites indicate higher strength of interfacial metal–ceramic bonding than after SPS processing as shown in Table 1.

Using nano-indentation as summarized in Table 2, the hardness of the interfaces in both the 10% and 20% SiC SPS samples is higher than for the HPT samples. This is attributed to the development of an interfacial Cu_3_Si phase and residual carbon. The presence of new phases/layers in the metal–ceramic interface zone may lead to an increased material volume response during indentation and a carbon structure will combine relatively high strength with a brittle character of deformation to therefore display higher resistance to compressive loading. Since the interfacial zone in the HPT samples lacks any additional phases/layers, it gives a lower response than in the SPS samples. An additional phenomenon is presented in Fig. 17 where the larger SiC particles in the SPS samples increase the possibility of indentation at their surface rather than indenting the interfacial zone. Conversely, the HPT interface consists of both large and fine SiC particles so that the possibility of SiC indentation is smaller and the recorded nano-hardness values may represent the true mechanical properties of the interfaces.

Both the SPS and HPT interfaces reveal higher hardness values as the SiC content increases. This effect is due to an enhanced dislocation density due to the thermal inconsistency between the Cu matrix and the ceramic reinforcement. During the cooling process, thermal stresses around the reinforcement are sufficiently large to generate plastic deformation in the matrix and this is especially anticipated in the contact bonding region [40]. These stresses induce dislocations within the area of the matrix–particle interface [41] which may accumulate with increasing reinforcement content. For the HPT interface, this effect is stronger due to the copper grain refinement and fragmentation process of the ceramic phase during processing, resulting in the formation of a more highly developed microstructure in the nanoscale. This will lead to a strengthening of the interface zone which is also responsible for the higher hardness of the metal–ceramic interface in both the SPS and HPT composites compared to the Cu matrix.

### The impact of HPT processing on the deformation and damage behavior of the overall system

**Fig. 20:**
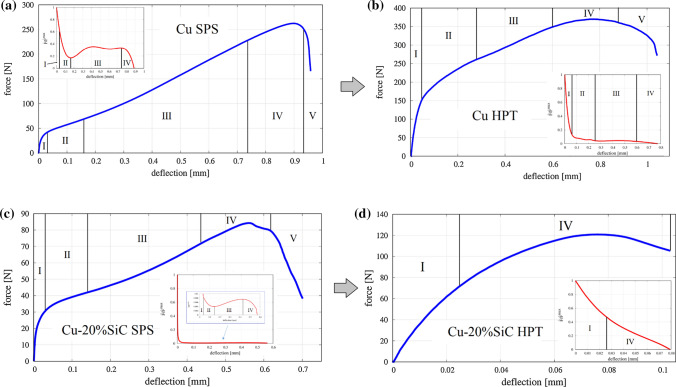
Representative force–deflection curves (with a normalized derivative of force in respect of deflection) of: **a** Cu SPS, **b** Cu HPT, **c** Cu–20%SiC SPS composite and **d** Cu–20%SiC HPT composite

Evaluation of deformation and damage behavior can be made from the investigation of force–deflection curves from small punch testing. Apart from the fact, they bring quantitative data from the test, such as an ultimate (maximum) force or maximum registered deflection, they allow qualitatively assess the behavior of the material. The shape of the F–D curve can be an important indicator confirming whether a material breaks in a ductile, brittle, or mixed manner. Similar analyses have been carried out in several works regarding the numerical and experimental investigation of SPT [20, 21]. Knowing the F–D curves for typical ductile and brittle material, we can assign studied SPS and HPT materials to each type of deformation and damage. To facilitate the determination of the F–D curve shape and its features, the parameter $\dot{F}$ was introduced related to curve slope and defined as the first derivative of registered force with respect of deflection—$\dot{F}=\mathrm{d}F/\mathrm{d}u$. Having this parameter normalized by its maximum value—$\dot{F}/{\dot{F}}^{\mathrm{max}}$, it is possible to catch any change of slope indicating the deformation character of the studied material. The evolution of the introduced parameter with the corresponding F–D curve of Cu and Cu–20%SiC composite processed by SPS and HPT routine has been shown in Fig. 20.

On the one hand, the large deformation brought a significant improvement of Cu mechanical strength, but on the other it did not considerably change the deformation character (Fig. 20a, b). At both F–D curves, we can highlight and denote specific stages defining the type of SPT deformation. In the initial stage of loading, the material deforms elastically and the deformation is influenced by Young’s modulus and yield strength. In the second stage, an elastoplastic transition occurs and plastic deformation begins to dominate on a much larger sample area. As the deformation covers the entire thickness of the sample range, the third stage begins. Here, it becomes the change in the main deformation mechanism from bending to membrane stretching in the sample. Generally, the moment of the transition is hard to catch on the F–D curve, however, it can be exposed by the use of the normalized $\dot{F}$ parameter. In the case of the SPS Cu sample, a vast increase of parameters is seen, which indicates a typical ductile type of deformation. The mentioned feature does not occur in the case of the second sample suggesting a slight transition of deformation type from ductile after SPS to mixed (brittle–ductile) after HPT (Fig. 20b). As confirmed by micro-tensile (Fig. 13) and nano-indentation study (Fig. 16), HPT reduces significantly the plastic range of deformation of Cu in the whole range of the material deformation.

The fourth stage begins with a decrease of the $\dot{F}$ parameter, which is related to a plastic instability with necking, crack initiation and propagation to failure. At the beginning of the fifth stage, we found macrocracks initiate [21] which is manifested by the descent of the F–D curve—steeper, limited for SPS and flatter, longer for the HPT sample.

The addition of 20% ceramic SiC reinforcement to the Cu matrix, apart from reducing tensile strength, keeps the character of the F–D curve in comparison to Cu SPS (Fig. 20c). The first stage of deformation reveals an increase of elastic response of the composite due to the presence of hard ceramic particles stiffening the entire system. The nearly imperceptible third stage shows the decrease of plastic contribution to deformation defining it as a mixed one (brittle–ductile). The fourth stage is accompanied by crack initiation on the metal–ceramic interface (Fig. 12c, e) as the most vulnerable and responsive component of SPS composite to mechanical tension (Fig. 13).

The higher volumetric content of SiC particles in composite, the more brittleness we may expect. As it was shown in Ref. [24], Cu matrix composites with 30%, 40% and 50% of ceramic content sintered by SPS breaks in a typical brittle manner. A similar effect can be obtained by the application of large deformation via HPT routine, which dramatically switches the character of deformation and damage manner of composite from mixed (after SPS) to a typical brittle one (after HPT) (Fig. 20d). In this case, it is possible to distinguish only two stages of deformation—the first one related to elastic response (I stage of SPT deformation) and the second one demonstrating the initiation and propagation of composite damage (IV stage of SPT deformation). Comparing to the SPS composite, I stage of the F–D curve of HPT composite points out the improved elastic response due to the several strengthening mechanisms of all composite components revealed by micro- and nano-level investigations. Strengthened Cu matrix (induced by grain refinement and lattice defects), the occurrence of nano-SiC particles (providing the Orowan effect, the load-bearing effect and load transfer between the matrix and the reinforcement) and interface zone between both phases (supported by an enhanced dislocation density strengthening mechanism) create the beneficial macroscopic system, which ensures the increased composite stiffness.

The transition between I and IV stages of SPT deformation can be identified by the change of curve slope of $\dot{F}$ parameter (Fig. 20d). Here, the initial cracking starts transgranular within the partially damaged and weaken SiC particles (Fig. 12d, f). As the composite damage propagates, we can expect the further composite components to join the process—intergranular cracking via metal–ceramic interface and Cu matrix in a transgranular manner.

## Summary

This research provides a first multiscale investigation of the mechanical properties of Cu samples and Cu matrix composite reinforced by SiC particles produced by the SPS technique and the combination of SPS and HPT routine. Global mechanical improvement of HPT materials has been achieved and demonstrated by the performance of small punch testing at macroscale. Both studied materials, Cu samples and Cu–SiC composites obtained much higher ultimate strength at RT and 350 °C.

Enhanced macroscopic strength is accompanied by the change of deformation and damage character. Micro-grained Cu sintered by SPS demonstrates a typical ductile type of deformation and damage with considerable plastic range. The inclusion of ceramic particles within the Cu matrix SPS decreases the plastic component of deformation making the deformation type a combination of brittle–ductile. HPT application of composite material impacts mostly the deformation and damage behavior so that it is a typical representation of the brittle type.

The change in dominant damage/failure mechanism from intergranular along the interfaces between the SiC particles and the Cu matrix (SPS) to the combination of inter- and transgranular through metal matrix and/or ceramic reinforcement (HPT) is caused by microstructural and hence mechanical performance of each composite component (metal matrix, ceramic reinforcement and metal–ceramic interface) at nano- and microscale. As microtensile strength test of the interfacial Cu–SiC bonding shows, the interface from HPT composites indicates higher ultimate tensile strength comparing to SPS one due to the reduction in defects, the annihilation of additional phase on boundary, and the influence of enhanced dislocation density strengthening acting mainly in the metal–ceramic contact zone.

Along with the evolution of Cu–SiC bonding, HPT processing considerably affects the structure, size and mechanical response of the ceramic reinforcement and metal matrix. The large and solid ceramic particles prevailing in SPS samples are largely replaced by a partially cracked and damaged SiC phase and a high fraction of ceramic nanoparticles indicating lower average nano-hardness during indentation testing at nanoscale. As the ceramic content increases, fragmentation becomes more effective, thereby lowering the numbers of large and damaged particles and raising the numbers of nanoparticles which is reflected in the gradual improvement of nano-hardness of the ceramic phase. Finally, by applying HPT processing, and thus obtaining a structure with ceramic nanoparticles, the Orowan mechanism becomes responsible for the enhanced nano-hardness in the metal matrix relative to Cu samples proven by nano-indentation testing. Likewise, the grain refinement and lattice defects of Cu samples brought on by HPT strengthen the material keeping ductility at a satisfactory level as the microtensile and nano-indentation results confirmed.
